# Intestinal Infections Among Febrile Hospitalized Patients in the Republic of Armenia: A Retrospective Chart Review

**DOI:** 10.1007/s10900-016-0174-x

**Published:** 2016-03-18

**Authors:** Eduard Zardaryan, Lusine Paronyan, Vahe Bakunts, Zaruhi Gevorgyan, Vigen Asoyan, Hripsime Apresyan, Alvard Hovhannisyan, Karo Palayan, Tinatin Kuchuloria, Robert G. Rivard, Christian T. Bautista

**Affiliations:** 1 The Nork Infectious Clinical Hospital, Yerevan, Armenia; 2 National Center for Disease Control and Prevention, Yerevan, Armenia; 3 U.S. Army Medical Research Institute of Infectious Diseases, Fort Detrick, MD USA; 4 Walter Reed Army Institute of Research, Silver Spring, MD USA

**Keywords:** Enteric, Etiology, Epidemiology, Surveillance, Armenia

## Abstract

In the past, several enteric outbreaks in 1996, 1998, 1999, and 2003 caused by *Salmonella typhi*, a Gram-negative bacterium, have occurred in Armenia. This study describes the demographic, epidemiological, and clinical characteristics of febrile hospitalized patients with intestinal infections in Armenia. Using a chart review study design, medical data from adult patients who were hospitalized at the Nork hospital during 2010–2012 were reviewed. A total of 600 medical charts were reviewed. Of these, 51 % were diagnosed with intestinal infections. Among these patients, 59 % had an intestinal infection of known etiology, with three main pathogens identified: *Salmonella* sp. (32 %), *Shigella* sp. (32 %), and *Staphylococcus aureus* (18 %). After controlling for the calendar year, age in years, and gender, patients detected with *Salmonella* sp. were more likely to reported the presence of a family member with similar signs or symptoms [odds ratio (OR) 9.0; 95 % CI 2.4–33.7] and the lack of a water tap at home (OR 3.9; 95 % CI 1.7–9.5). Evidence indicates that *Salmonella* sp., *Shigella* sp., and *S. aureus* as the most common etiologies reported among febrile hospitalized patients. A high percentage of patients had intestinal infections of unknown etiology; thus, improvement in laboratory capacity (enabling more advanced tests, such as polymerase chain reaction) would increase the identification of the enteropathogens causing disease in Armenia.

## Introduction

In many developing countries, intestinal infections represent the main source of morbidity and pose a significant challenge to public health [1]. Several epidemiological studies have reported individual-level associations between intestinal infections and risk factors such as contaminated food, non-potable water, close contact with animals, and international travel [2–4].

The Republic of Armenia, a country in the South Caucasus region, has an estimated population of 2.9 million, with 65 % living in urban areas. Agriculture plays an important role in the national economy [5]. After Armenia gained independence from the Soviet Union in 1991, the economy and the health care system deteriorated. According to the European Observatory on Health Systems and Policies, Armenia has had since 2010 a strong economic growth, which benefited public health; but the health system remains concentrated in Yerevan, the capital city [6]. Further, most hospitals, pharmacies, and dental services have been privatized in Armenia.

The availability of information on the burden of specific infectious diseases in Armenia is limited. Data from the Institute for Health Metrics and Evaluation show that the main causes of premature death in 2010 were ischemic heart disease, stroke, lung cancer, and diabetes [7]; diarrheal diseases were ranked 27th. In the past, several outbreaks of intestinal infections in 1996, 1998, 1999, and 2003 caused by *Salmonella typhi*, a Gram-negative bacterium, have occurred in Armenia. Contaminated drinking water in households was associated with the outbreaks [8]. During the most recent outbreak of intestinal infection, which took place in June 2011 in Nubarashen, an administrative district of the city of Yerevan, 57 patients were hospitalized at the Nork Infectious Clinical Hospital (hereafter, “Nork hospital”) (unpublished data). Based on field investigations, the public water pump was suggested as the source of the outbreak. Since 1956, the Nork hospital has been Armenia’s main medical institution specializing in the diagnosis and treatment of infectious diseases. In Armenia, during 2005–2008, household access to piped water increased from 65 to 97 % in urban areas and from 36 to 70 % in rural areas [9]. According to a 2006 World Health Organization report, an estimated 99 and 80 % of urban and rural residents, respectively, have access to drinking water in Armenia [10]. In addition, drinking water quality is systematically monitored across the country.

This report aimed to extent the previous findings to more detailed information on the morbidity of intestinal infections among febrile hospitalized patients at the Nork hospital between 2010 and 2012 [11]. To our knowledge, this is the first study of its kind in Armenia.

## Materials and Methods

### Study Setting, Participants, and Data Collection

Briefly, we conducted a retrospective chart review study among patients hospitalized at the Nork hospital between January 2010 and December 2012 [11, 12]. The inclusion criteria for this study included: (1) adult patients (≥18 years old) with an axillary temperature at hospital admission of 38 °C or higher, and (2) a duration of hospitalization of at least 24 h. Based on the official medical charts stored at the Nork, a standardized questionnaire was developed in English and translated into Armenian. This data-instrument collected patient information on demographics (e.g., gender and age), hospitalization (e.g., preliminary diagnosis), clinical signs and symptoms (e.g., fever), epidemiological exposure (e.g., exposure to animals), physical exams, treatment before and during hospitalization, and laboratory test results.

The Nork hospital study team performed medical record data extraction and patient names were not collected. The study questionnaires were entered into an Epi Info database at the National Center for Disease Control and Prevention. For data entry quality control, a random 1 in 5 sample of the questionnaires was double entered, and discrepancies with the original data were identified and corrected. After completing the data entry, questionnaires were securely stored in the Nork hospital archive. The final clinical diagnosis was classified according to the International Classification of Diseases, version 9. For determining the number of charts needed for this study, the Nork study team indicated that, of the 5000–6000 patients admitted to the hospital annually, approximately 250–300 patients met inclusion criteria. Once identified, patients that met the study inclusion criteria, we applied a convenience sampling approach (non-probability sampling) to identify 200 patient medical charts per year for data extraction, analysis, and reporting. In order to study seasonal variations or other changes over time, we extracted (where available) on average the first 16–17 medical charts per month. In total, over the study period (2010–2012), 600 patient medical charts were reviewed.

At the Nork hospital, the laboratory detection of *Shigella* and *Salmonella* spp. are done by stool culture. Rotavirus, *E. coli* (pathogenic), and *Campylobacter* sp. are diagnosed using immunoenzymatic assays (IEA) in stools, and *Entameba histolytica* is diagnosed by both IEA and by stool microscopy. Detection of *Staphylococcus aureus* bacteria is done using culture methods. At the hospital, infection with *S. aureus* is, defined as a culture bacterial overgrowth of >10^4^ colony-forming units per ml and typical clinical symptoms (e.g., nausea, vomiting, and diarrhea).

The study protocol was reviewed and approved by the Nork Infectious Clinical Hospital, National Center for Disease Control and Prevention, U.S. Army Medical Research Institute of Infectious Diseases (FY13-20), and Walter Reed Army Institute of Research (WRAIR #2098). Because this was a retrospective study, informed consent was waived.

### Statistical Analysis

Categorical variables were compared using the Pearson Chi square or Fisher’s exact test. For quantitative variables, means and standard deviations (SD) were reported and the Student’s *t* test or Mann–Whitney U test was used to compare means. Three or more means were compared using the nonparametric Kruskal–Wallis test. Associations expressed as odds ratios (OR) with 95 % confidence intervals (CI), were computed using multinomial logistic regression analyses. Reported *p* values were two-sided, and *p* values less than 0.05 were considered statistically significant. Analyses were carried out using Epi Info version 3.5.3 (Centers for Disease Control and Prevention, Atlanta, GA, USA) and IBM SPSS Statistics for Windows, version 20.0 (IBM Corp., Armonk, NY, USA).

## Results

During the study period, medical charts of 600 patients were reviewed. Of these, 305 (51 %) patients were diagnosed with an intestinal infection: 94 (43 %) in 2010, 105 (52 %) in 2011, and 106 (60 %) in 2012. Among this group of patients, all of them had a stool culture, and in 59 % (179 of 305) detected the bacterial pathogenes associated with intestinal infection: 64 % (60 of 94) in 2010, 57 % (60 of 105) in 2011, and 56 % (59 of 106) in 2012.

Among patients with an intestinal infection of known etiology (n = 179, Table 1), the mean age was 36.1 years; 43 % were males, 84 % were Yerevan residents, and 36 % of the hospital admissions occurred between the months of June and August. Our analysis revealed that these demographic characteristics were similar to patients with intestinal infection of unknown etiology, except for age in years. Hospitalized patients with unknown etiology were older than that of patients with known etiology (39.8 vs. 36.1 years; *p* = 0.028). No other variables differed significantly between the two groups of patients.

**Table 1:** Demographic, clinical, and epidemiological characteristics of patients with intestinal infections of unknown and known etiology at the Nork hospital, 2010–2012

Feature	Unknown etiology n (%)	Known etiology n (%)
No. of patients	126	179
*Demographic data*		
Age in years, average (SD)	39.8 (17.4)	36.1 (17.5)
Male gender	53 (43)	75 (43)
Armenian citizenship	123 (98)	175 (98)
Yerevan residence	106 (84)	149 (84)
June–August admission	44 (35)	65 (36)
*Clinical data*		
Signs		
Fatigue	96 (76)	95 (53)
Diarrhea	84 (67)	75 (42)
Nausea/vomiting	87 (69)	84 (47)
Shaking/rigors	52 (41)	46 (26)
Abdominal pain	78 (62)	79 (44)
Headache	20 (16)	16 (9)
*Physical examination*		
Abdominal tenderness	107 (85)	154 (86)
Pallor	82 (65)	127 (71)
Abdominal distention	51 (40)	71 (40)
Pharyngeal injection	24 (19)	38 (21)
Antibiotic treatment before hospital admission	26 (21)	24 (13)
Chloramphenicol	12 (46)	9 (37)
Antibiotics at hospital	89 (71)	140 (78)
Ciprofloxacin	81 (91)	123 (88)
Trimethoprim–sulfamethoxazole	6 (7)	19 (13)
Average duration of hospitalization in days ± SD	4.4 ± 2.1	5.0 ± 2.2
*Epidemiological data*		
Water tap at home	83 (66)	118 (66)
Undercooked meat products	23 (18)	34 (19)
Unpasteurized dairy products	3 (2)	6 (3)
Family member with similar signs	9 (7)	24 (13)
Traveled outside Armenia	4 (3)	4 (2)

*SD* standard deviation. Denominator may vary due to missing data

Among patients with an intestinal infection—whether of known or unknown etiology—the most frequently reported clinical signs and symptoms after fever were fatigue, nausea/vomiting, abdominal pain, and diarrhea. Other signs or symptoms, such as headache, unusual bleeding, stiff neck, rash, sore throat, joint pain, skin lesions, and pain behind eyes, were reported in few patients in both groups (less than 10 %). The signs most frequently reported via physical examination were abdominal tenderness, pallor, abdominal distention, and pharyngeal injection. The two groups were similar in their distributions of clinical signs/symptoms and physical examination results (Table 1).

**Fig. 1:**
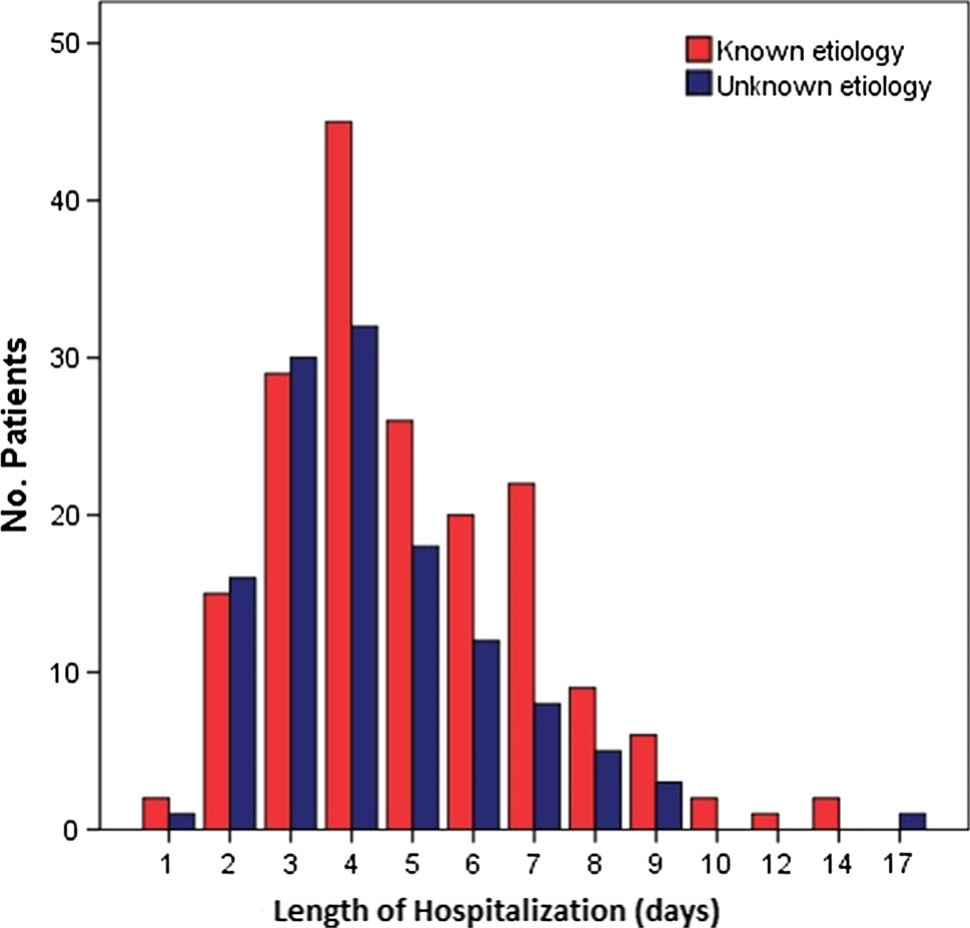
The distribution of hospitalization durations for both groups of patients

Our analyses showed that 13 and 21 % of patients with known and unknown etiology, respectively, received antibiotic treatment before hospital admission, with chloramphenicol the antibiotic most frequently used in both groups. Patients with unknown etiology were more likely to have received antibiotic treatment before hospital admission, although this association was not significant (OR 1.8; 95 % CI 0.9–3.3; *p* = 0.065). During hospitalization, 78 % of patients with an intestinal infection of known etiology were treated with antibiotics. This percentage was higher than that of patients with an intestinal infection of unknown etiology (71 %), although the difference was not significant (*p* = 0.128). Ciprofloxacin was the drug most commonly used among both groups of patients at the Nork. The mean duration of hospitalization was longer for patients with infections of known etiology than for patients with infections of unknown etiology (5.0 vs. 4.4 days, *p* = 0.025, Fig. 1).

Regarding epidemiological data, among patients with an intestinal infection of known etiology, 66 % reported that they had a water tap at home, 19 % had consumed undercooked meat products, 13 % had a family member with similar signs or symptoms, and 3 % had consumed unpasteurized dairy products. Similar percentages were reported by patients with an intestinal infection of unknown etiology, except that these patients were less likely to have a family member with similar signs or symptoms (7 vs. 13 %), although the difference that did not reach statistical significance (*p* = 0.118).

Overall, the three main pathogens among patients with known etiology were *Salmonella* sp. (n = 57, 32 %), *Shigella* sp. (n = 57, 32 %), and *S. aureus* (n = 32, 18 %). When stratified by year (2010, 2011, and 2012, respectively), the number of hospitalizations caused by *Salmonella* sp. were 13, 20, and 24; the number caused by *Shigella* sp. were 28, 19, and 10; and the number caused by *S. aureus* were 10, 14, and 8. These pathogens were more prevalent in women, such that 66 % of patients detected with *Shigella* sp. were women, as were 52 % of those with *Salmonella* sp., and 62 % of those with *S. aureus*. Patients older than 27 years made up the majority of those known to have *Shigella* sp. (56 %) and *Salmonella* sp. (51 %), but not *S. aureus* (44 %). Other pathogens, such as enterohemorrhagic *E. coli*, *Campylobacter* sp., *Entameba histolytica*, and rotavirus, were reported, but to a lesser extent.

We performed a comparative analysis of selected characteristics among hospitalized patients detected with *Salmonella* sp., *Shigella* sp., and *S. aureus*. According to this analysis, patients detected with *Shigella* sp. were more likely to report fatigue, to have received antibiotic treatment at the hospital, and to have a water tap at home (Table 2). In contrast, patients detected with *Salmonella* sp. were more likely to report nausea/vomiting and abdominal distention, to spend more time in the hospital, and to have a family member with similar signs and symptoms. In contrast, patients detected with *S. aureus* were more likely to be Yerevan residents, to have been hospitalized between June and August, and to report shaking/rigors compared with patients detected with *Salmonella* or *Shigella* spp.

**Table 2:** Selected demographic and epidemiological characteristics of patients detected with *Shigella* sp., *Salmonella* sp., and *S. aureus* at the Nork hospital, 2010–2012

Feature	*Shigella* sp.	*Salmonella* sp.	*S. aureus*	*P* value
	n (%)	n (%)	n (%)	
No. patients	57	57	32	
Residence, Yerevan	46 (81)	45 (79)	32 (100)	0.021
Seasonality (admitted Jun–Aug)	21 (37)	36 (63)	21 (66)	0.006
Fatigue	57 (100)	49 (86)	31 (97)	0.006
Nausea/vomiting	46 (81)	54 (95)	25 (78)	0.040
Shaking/rigors	17 (30)	27 (47)	20 (62)	0.014
Abdominal distention	19 (33)	30 (53)	7 (22)	0.010
Antibiotics at hospital	52 (91)	48 (84)	21 (66)	0.008
Duration of hospitalization in days, mean ± SD	5.2 ± 2.5	5.7 ± 2.4	3.9 ± 1.6	0.011
Water tap at home	47 (82)	31 (54)	23 (72)	0.005
Family member with similar signs	4 (7)	15 (26)	0 (0)	<0.001

*SD* standard deviation. Denominator may vary due to missing data

Logistic analysis revealed that, after controlling for the calendar year, age in years, and gender, and using patients detected with *Shigella* sp. as the reference group, the presence of a family member with similar signs or symptoms (OR 9.0; 95 % CI 2.4–33.7; *p* = 0.001) and the lack of a water tap at home (OR 3.9; 95 % CI 1.7–9.5; *p* < 0.001) were significant predictors for patients detected with *Salmonella* sp. Of the 57 patients detected with *Salmonella* sp., 26 % had a family member with similar signs or symptoms, and 44 % lacked a water tap at home.

## Discussion

In this study, intestinal infections were the main causes of hospitalization among febrile patients at the Nork hospital between 2010 and 2012, with *Salmonella* sp., *Shigella* sp., and *S. aureus* the most commonly reported etiologies, as well as 41 % of patients had an intestinal infection of unknown etiology.

Our analyses indicated that, during the study period, the percentage of hospitalizations caused by intestinal infections increased from 43 % in 2010 to 60 % in 2012, and the percentage of patients with an intestinal infection of known etiology decreased from 64 % in 2010 to 56 % in 2012. In Armenia, the most recent outbreak of intestinal infection occurred in June 2011 in Nubarashen, one of the twelve districts of Yerevan city. During the 2011 outbreak, the Nork hospital, as the referral clinic for care, hospitalized 57 patients affected by the outbreak. It was not possible to determine, from the medical charts, the percentage of hospitalizations linked to the 2011 outbreak; thus, our 2011 estimates should be interpreted with caution. Moreover, the reasons for these trends are not fully clear from our study data. Nevertheless, after excluding 2011 data, we found that the percentage of hospitalizations associated with intestinal infections in 2012 was significantly higher than that in 2010 (60 vs. 40 %, *p* < 0.001). One might speculate that a true increase in hospitalizations caused by major bacterial enteric pathogens occurred at the Nork hospital in recent years.

Interestingly, demographic, epidemiological, and clinical characteristics were similar between patients with intestinal infections of known and unknown etiology, with the exceptions of age and duration of hospitalization. Regarding duration of hospitalization, patients with infections of known etiology had, on mean, a longer stay than patients with infections of unknown etiology. We also found that medical complication was associated with a longer stay in hospital. None of the patients with infections of unknown etiology had a medical complication. However, among patients with infections of known etiology, those with medical complications were hospitalized longer, on average, than those who did not experience medical complications (6.3 vs. 4.9 days, *p* = 0.095). Although this difference did not reach statistical significance, medical complications contributed to a longer duration of hospital stay that was approximately 34 h longer than patients without complications. This is consistent with previously published studies [13–15]. After excluding patients with medical complications, the duration of hospitalization among patients with infections of unknown etiology was similar to that of patients with infections of known etiology (4.4. vs. 4.9 days).

This chart review study revealed that, at the Nork hospital during 2010–2012, the most common pathogens associated with intestinal infection—when etiology was known—were *Salmonella* sp., *Shigella* sp., and *S. aureuss*, which together accounted for 82 % of all pathogens. Each of these bacterial pathogens has a worldwide distribution, with incidence rates and temporal patterns in different regions of the world that vary according to the level of exposure and sanitation [16]. However, two important features in the epidemiology of these bacterial pathogens stand out. First, they have a consistent seasonal pattern, with most infections occurring during the warmest periods of the year [17]. This is probably due to the rapid growth of bacteria at warmer temperatures and the resulting potential for foodborne illness [18]. Our findings were consistent with these seasonal patterns. We found that 37, 63, and 66 % of patients detected with *Salmonella* sp., *Shigella* sp., and *S. aureus*, respectively, occurred between June and August. In Yerevan city, the daytime temperatures during summer can reach around 40 °C (104 °F).

A second notable set of features in the epidemiology of these pathogens are the mechanisms and routes by which they are transmitted. *S. aureus* transmits through the consumption of contaminated food. In contrast, *Salmonella* and *Shigella* spp. transmit via multiple routes—primarily person-to-person or through contaminated water or food [19–21]. Due to the limited epidemiological information recorded on the medical charts, it was not possible to study the routes of transmission for these bacterial pathogens. Despite this limitation, an interesting finding was the association between patients with *Salmonella* sp. and the presence of family members with similar signs or symptoms. We suggest that the Nork hospital encourage sick family members of patients with salmonellosis—as well as other intestinal infections—to seek medical attention, especially if their signs or symptoms are similar [22]. In addition, transmission routes may underlie the association between salmonellosis cases and the lack of a water tap at home. In developing countries where many households lack access to tap water in the home, waterborne transmission is a common route for most enteropathogens, including *Salmonella* sp. Further research is required to describe the individual and community-level factors for diarrheal disease transmission in Armenia.

According to the U.S. National Outbreak Reporting System, the etiology was unknown in 40 % of outbreaks of acute gastroenteritis during 2009–2010 [23]. In our study, 4 out of 10 hospitalized patients diagnosed with intestinal infection had an unknown etiology. Based on available study data, a possible explanation of the large percentage of patients with unknown etiology in our study may be due to the use of antibiotics before admission at the Nork hospital. However, other factors might also contribute to unknown etiology in Armenia; this is a topic that needs more investigation. From a clinical perspective, it is important to determine the cause of enteric infections, not only for direct clinical treatment, but also to detect outbreaks and to decrease morbidity and mortality in the population.

In Armenia and in many developing countries, a stool culture is the only laboratory test for diagnosis of enteropathogens, and thus, our findings should be taken with caution. On the one hand, culture of stool samples after beginning treatment may limit detection. In our study population, a high percentage of patients with unknown etiology received antibiotic treatment before hospital admission. On the other hand, a positive stool culture does not necessarily indicate the existence of *S. aureus*, which is not a well-known etiology of gastroenteritis. Therefore, further studies with molecular laboratory methods such as polymerase chain reaction based test [24], which compared to conventional methods is highly sensitive and detect low levels of enteropathogens, can significantly provide a better understanding of the etiology of intestinal infections in Armenia. Despite this laboratory limitation, to our knowledge, this is the first report that describes the epidemiological and clinical characteristics of intestinal infections among febrile hospitalized patients in the Republic of Armenia.

## Conclusion

At the Nork hospital between 2010 and 2012, half of the febrile hospitalized patients were diagnosed with intestinal infections and, among those patients with infections of known etiology, *Salmonella* sp., *Shigella* sp., and *S. aureus* were the most common pathogens. It is necessary the implementation of an integrated food chain surveillance system—in which samples from humans, animals, and food are collected and analyzed by molecular laboratory methods that include serotyping—to improve the detection and provide useful epidemiological information on the burden of intestinal infections in Armenia.
